# Anaesthesia Management of A Patient with Airway Obstruction Caused by Prosthetic Vascular Graft Invasion into the Tracheal Lumen

**DOI:** 10.4274/TJAR.2024.241627

**Published:** 2024-10-30

**Authors:** Serdar Demirgan, Gülçin Karacan, Sezen Kumaş Solak, Burcu Akyüz, Hakkıcan Akpolat, Ayşin Selcan

**Affiliations:** 1 University of Health Sciences Turkey, Bağcılar Training and Research Hospital, Clinic of Anaesthesiology İstanbul, Turkey

**Keywords:** Anaesthetic management, bronchoscopy, difficult airway, prosthetic vascular graft, tracheal obstruction

## Abstract

Primary intratracheal masses causing luminal obstruction are relatively rare, posing a challenge for anaesthesiologists in airway management. This case report describes a distinctive airway management approach in a 71-year-old female patient with an aorta-carotid artery bypass graft that significantly obstructed the trachea.

The patient presented with worsening shortness of breath, and a thoracic computed tomography scan revealed a 19.2 mm×9.9 mm×19.3  contrast-enhancing mass penetrating the right anterolateral tracheal wall, resulting in 80% occlusion of the tracheal lumen. Awake fiberoptic bronchoscopy (FOB)-guided nasotracheal intubation was performed following topical upper airway anaesthesia, with the patient positioned at a 30º head-up angle and slight right-up tilt to minimize discomfort. A 6.0 mm ID cuffed endotracheal tube was successfully placed under fiberoptic guidance distal to the intratracheal vascular graft but proximal to the carina. Intratracheal masses can lead to severe tracheal obstruction followed by progressive airway obstruction, which can be life-threatening when effective ventilation cannot be established after the induction of general anaesthesia. We recommend the use of awake FOB-guided intubation in such cases. Additionally, contingency plans should be prepared and meticulously prepared in the event of intubation or ventilation failure.

## Introduction

Tracheal masses are extremely rare; however, they can result in various complications, depending on their growth rate, duration, and degree of obstruction.^1^ Severe airway obstruction is generally defined as occlusion of >70% of the tracheal lumen.^2^ Obstruction can occur due to external compression or the presence of masses within the trachea. Both conditions can pose challenges for airway management, especially during the perioperative period.^3^ Anaesthesiologists face particular difficulties in the perioperative management of patients with tracheal masses.^3^ The anaesthetic approach requires careful planning, especially when preoperative assessment indicates difficulty. We present a unique case of safe and successful airway management via awake fiberoptic bronchoscopy (FOB)-guided nasotracheal intubation in a patient with an aortic-carotid artery bypass graft that was invading and significantly obstructing the trachea.

## Case Report

### Medical History

**Figure 1:**
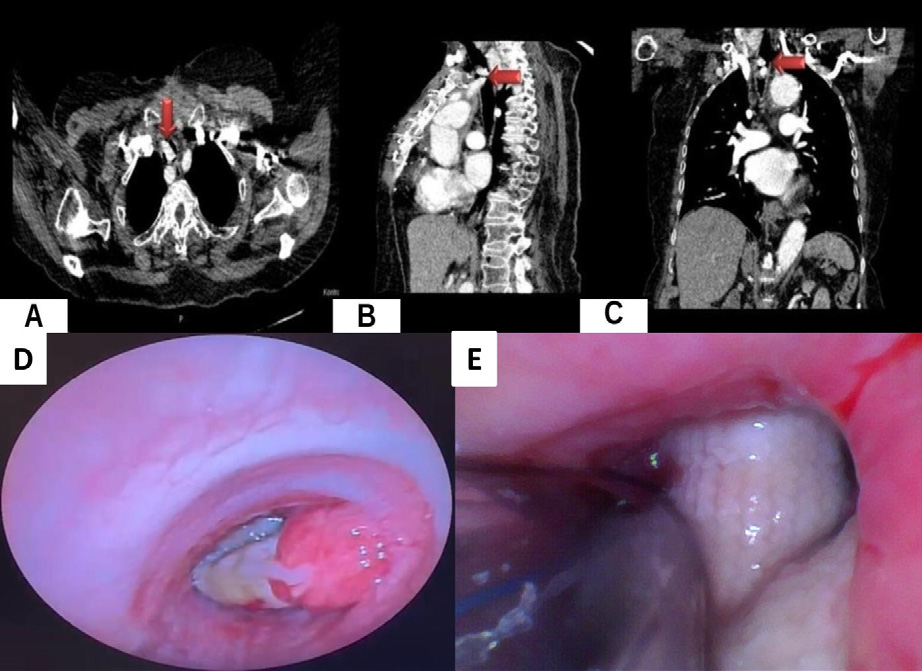
A-C) Contrast-enhanced computed tomography of the chest shows an aorta-carotid bypass graft perforating the right anterolateral wall of the trachea, occluding the tracheal lumen, D) Preoperative video-bronchoscopy showing intratracheal aorta-carotid bypass graft significantly obstructing the tracheal lumen, E) Standard tracheal tube (6.0 mm ID) passing over the prosthetic vascular graft in the trachea.

The patient provided consent for the clinical information pertaining to the case to be published in a medical journal. The patient was a 71-year-old with no history of smoking and was taking a calcium channel blocker for hypertension and an inhaler beta-agonist for asthma. In 2018, she underwent surgery for type 2 aortic dissection according to the DeBakey classification. In 2022, the patient was admitted due to a pulsatile mass extending to the skin at the site of the sternum defect. Further examination revealed saccular aneurysmatic dilation at the arcus aorta. As a result, the patient underwent ascending aortic replacement, hemiarch replacement, and right-left debranching bypass. Subsequently, she returned to our hospital due to increasing respiratory distress during the 21-month postoperative period. Thorax computed tomography of the patient revealed a 19.2 mm×9.9 mm×19.3 contrast-enhancing mass perforating the right anterolateral wall of the trachea, occluding the tracheal lumen by 80% (Figure 1A-C). Preoperative FOB showing a hole in the anterolateral tracheal wall with invasion of the tracheal lumen by the prosthetic vascular graft (Figure 1D). The patient was scheduled for a revision of the aorta-carotid artery bypass graft and tracheal resection with primary anastomosis.

### Anaesthesia Management

Standard endotracheal intubation was considered unfeasible and extremely risky. The primary aim was to perform fiberoptic intubation of the patient’s trachea. Plan B involved performing airway rescue using an extraglottic airway device. The patient was categorized as American Society of Anesthesiologists IV. Her body mass index was approximately 21 kg m^-2^ (height 155 cm, weight 50.3 kg). The patient’s airway was evaluated as Mallampati class II. The preoperative hemoglobin level was 10 g dL^-1^, and the hematocrit value was 31. Other laboratory tests exhibited normal results.

After the patient entered the operating room, standard non-invasive monitoring was initiated. Invasive arterial pressure was monitored using left radial artery catheterization. The nasotracheal route was prepared by applying 4% lidocaine for anaesthesia, and “conscious sedation” was achieved using midazolam (3 mg intravenous) and infusion of remifentanil (0.05-0.1 µg kg^-1^ min^-1^). Awake FOB-guided nasotracheal intubation was performed. Under fiberoptic guidance, a 6.0 mm ID cuffed endotracheal tube was placed distal to the intratracheal vascular graft but proximal to the carina (Figure 1E). The patient was then anaesthetized and paralyzed with an injection of propofol (1 mg kg^-1^, fentanyl 2 µg kg^-1^, and rocuronium (0.6 mg kg^-1^).

The patient underwent tracheal resection and reconstruction, as well as aorto-carotid artery re-interposition. Anaesthesia was maintained with FiO_2_ 0.5, sevoflurane 1-2%, and remifentanil infusion at 0.05-2 µg kg^-1^ min^-1^. The procedure lasted for 374 minutes. During the perioperative period, patients with a blood loss of 2,000 cc received 4 units of packed red blood cells and 1 unit of fresh frozen plasma transfusion. The patient also received a crystalloid infusion of 3,000 cc and an 800 cc urine output.

### Postoperative Management

Extubation was not performed at the end of the operation due to the patient’s initial partial carbon dioxide pressure of 75 mmHg, indicating hypercapnia. In addition, persistent hypoxemia was observed during surgery. The patient was then transferred to the intensive care unit (ICU) in an intubated state. On postoperative day 7, the patient’s mechanical ventilation parameters and clinical conditions improved. Her oxygen saturation improved to 97% with an FiO_2_ of 50% and a positive end‐expiratory pressure of 5 cm H_2_O. She had stable vital signs. She was extubated on postoperative day 7 and discharged from the ICU on postoperative day 12. The preoperatively positioned 6.0 mm ID cuffed endotracheal tube facilitated continued mechanical ventilation during surgery and until extubation. The patient experienced an uncomplicated recovery and was discharged from the hospital 39 days later.

## Discussion

Primary intratracheal masses causing luminal obstruction are relatively uncommon, and they pose a therapeutic challenge for anaesthesiologists during airway management.^4^ Several processes are responsible for tracheal obstruction. These etiologies include benign and malignant primary tracheal tumors, extrinsic compression of the airway, postintubation or posttracheostomy tracheal stenosis, stenosis related to airway stents, inflammatory diseases (e.g., sarcoidosis, granulomatosis with polyangiitis, relapsing polychondritis), and dynamic airway narrowing (tracheobronchomalacia).^2^ Tracheal obstruction due to the thyroid and parathyroid glands has been reported.^5, 6^ However, tracheal stenosis resulting from a prosthetic vascular graft within the trachea has not been previously described. This unique case involved erosion of the anterolateral tracheal wall over time by the aorta-carotid artery bypass graft, resulting in its entry into the tracheal lumen. The possible etiology of this condition is that the prosthetic vascular graft is longer than it should be, and chronic irritation and erosion occur as a result of the graft’s contact with the tracheal wall.

Patients with tracheal stenosis may present with dyspnea on exertion, shortness of breath, stridor, or wheezing, with symptoms lasting several years.^7^ Often, they remain asymptomatic until approximately two-thirds of the tracheal diameter is occluded, potentially leading to a life-threatening condition.^8^ In our case, 80% occlusion of the tracheal lumen aggravated dyspnea, prompting a surgical decision to remove the intratracheal mass. Patients experiencing respiratory distress due to an intratracheal mass are frequently initially misdiagnosed. In our case, the patient’s pre-existing asthma diagnosis delayed the identification of a tracheal mass. The patient’s unresponsiveness to bronchodilator treatment was key to diagnosis. Similarly, a case of intratracheal schwannoma misdiagnosed as asthma has been reported in the literature.^9^ Failure to respond to standard treatment should prompt consideration of alternative diagnoses.

Intratracheal masses can lead to severe tracheal obstruction followed by progressive airway obstruction, which can be life-threatening when effective ventilation cannot be established after the induction of general anaesthesia.^3^ Consequently, intraoperative airway management in patients with endotracheal mass or severe airway stenosis poses a significant challenge for anaesthesiologists. In our case, conventional tracheal intubation was found to pose a significant risk to the patient. The most perilous scenario was the misplacement of the tracheal tube into the mediastinum during intubation performed without FOB guidance, in which the tube exited the tracheal defect. Another significant risk was the potential for the tracheal tube to displace the prosthetic vascular graft into the lower trachea, leading to complete airway obstruction. Both scenarios posed substantial risks to the patient’s life; hence, awake FOB-guided intubation was performed. Additionally, various perioperative airway management strategies were devised to address expected and unexpected conditions, such as intubation and ventilation failure. Instruments for cardiopulmonary bypass and extraglottic airway devices were made available. On the other hand, in cases of upper tracheal masses, preoperative tracheostomy under local anaesthesia can be an alternative to FOB-guided endotracheal intubation.^1^ However, for masses in the middle and lower tracheal regions, FOB-guided intubation appears to be the only alternative.

In the present case, we preferred a standard 6.0 mm ID cuffed endotracheal tube for tracheal intubation. The micro laryngeal surgery tube, an alternative to the conventional tube for cases in which the tracheal tube cannot bypass the mass, is longer and softer than the standard tracheal tube, enabling it to reach the carina while minimizing trauma to the endotracheal mass.^4^ However, unlike tracheal tumors, the prosthetic vascular graft embedded in the trachea was neither rigid nor posed a risk of bleeding during tube passage. Therefore, we selected the largest tracheal tube that we thought would not cause a serious decrease in blood flow past the graft. The preoperatively positioned tracheal tube facilitated continued mechanical ventilation during surgery and until extubation on postoperative day 7.

Early extubation is recommended to prevent tension on the suture line caused by the tracheal tube cuff and the potential adverse effects of mechanical ventilation.^10^ Although early extubation was not achieved due to persistent postoperative hypoxemia, the patient was successfully extubated without complications on postoperative day 7. The patient experienced an uncomplicated recovery and was discharged from the hospital 39 days later.

In summary, before the procedure, the risks associated with airway management techniques and the anaesthetic approach must be evaluated due to severe airway obstruction caused by intraluminal tracheal masses. We recommend the use of awake FOB-guided intubation in such cases. Furthermore, alternative plans should be formulated and meticulously prepared in case of intubation or ventilator failure. Effective communication and collaboration between healthcare providers are also crucial for ensuring successful outcomes.
